# Description of a new, unusual species of *Diestostemma* Amyot & Serville (Hemiptera, Cicadellidae) from Ecuador

**DOI:** 10.3897/zookeys.908.38477

**Published:** 2020-02-03

**Authors:** Stuart H. McKamey

**Affiliations:** 1 Systematic Entomology Laboratory, PSI, Agricultural Research Service, U.S. Department of Agriculture, c/o National Museum of Natural History, P.O. Box 37012, Washington, D.C. 20013, USA National Museum of Natural History Washington United States of America

**Keywords:** Auchenorrhyncha, Neotropical, new species, Proconiini, sharpshooter, taxonomy

## Abstract

*Diestostemmabicristata***sp. nov.**, is described from Napo Province, Ecuador. It is unusual for the genus in lacking a visible white powdery covering, having raised, weakly reticulate veins on the forewing, a short metathoracic femur (half length of the metathoracic tibia), and is the only species with a double-crested pronotum.

## Introduction

Sharpshooters are members of the cosmopolitan Cicadellinae, the third largest leafhopper subfamily, with over 2200 valid species among 327 genera. Sixty-two of these genera, and 425 species, belong to the New World tribe Proconiini (Young 1968, Cavichioli and Sakakibara 1989, Mejdalani and Emmrich 1998, Marucci et al. 2002, Godoy 2005, Rakitov and Godoy 2005, McKamey 2007), which includes the genus *Diestostemma* Amyot & Serville.

Species of the genus *Diestostemma* occur from Mexico to Argentina and are distinguished by an apical scar at the apex of the head (also present in *Proconia* Le Peletier & Serville) and a digitate posterior process on the pronotal lateral lobe (the portion behind the eye), both of which are present in the new species described here. Schmidt (1910) was the first to revise the entire genus, treating 17 species, 11 of which were described as new. Subsequently, Young (1968) revisited the genus, adding eight new species, placing seven of the previously described species (including three of Schmidt’s) in synonymy, and pulling in four species from various genera. Wilson et al. (2009) provided high resolution images of 31 of the 32 species described at the time. Mejdalani and Silva (2010) provided the first detailed description of the female terminalia of a *Diestostemma* species. Most recently, Pinto et al. (2017) revised the *D.bituberculatum* (Signoret) complex, which all have two dorsally directed protrusions on the pronotum. Their description of four new species brought the total valid species in the genus to 36. As far as known, nymphs have a uniform morphology (as in Fig. 5).

## Materials and methods

In providing distribution data of the holotype, quotation marks separate labels and a vertical line separates lines on a label.

Terminology for general morphology was based on Young (1968), and Mejdalani (1998), while leg chaetotaxy follows Rakitov (1998).

A Leica MZ12 stereomicroscope, with an ocular micrometer, was used to examine structures and to determine ratios between smaller distances. The larger dimensions of the head and pronotum were measured with a manual 5mm micrometer and body length was measured using a digital micrometer.

The abdomen was detached, macerated in a warmed, 10% KOH solution for 24 hours at room temperature, bathed in water, then acetic acid to stop the reaction. After dissection, structures were stored in a glass microvial containing glycerin and pinned beneath the specimen.

Images were taken with a Canon EOS 5Dsr camera with an adjustable Canon MP-E 65 mm lens. Photos were taken using Capture One Pro version 10.1.2, 64 bit, build 10.1.2.23 imaging software, aided by CamLift version 2.9.7.1. The specimen was lighted using two adjustable Dynalite MH2050 RoadMax flash heads, each attached to a Manfrotto 244 arm. The light was diffused using a simple, lampshade-style cone of translucent paper between the specimen and light sources. After individual “slices” were photographed, they were compiled into a single, composite image using Zerene Stacker - USDA SI-SEL Lab Bk imaging system, version 1.04, build T201706041920. Stacked images were enhanced and edited in Adobe Photoshop CSS Extended version 12.0. The scale bar (in Fig. 2) was generated through Photoshop directly from the metadata of the photo.

## Results

### Diestostemma bicristata sp. nov.

Taxon classificationAnimaliaHemipteraCicadellidae

B30D3112-CFAD-5D6E-AB58-780BE45541FB

http://zoobank.org/457964A9-0AAE-47DB-8A54-0DCB296BC168

Figs 1–4 , 5–12

#### Diagnosis.

A small narrow species with a long head, pronotum with two long, weakly converging raised ridges, forewing with extra crossveins but only weakly reticulate. Aedeagus of male in lateral view with gradually recurved basiventral process.

#### Material examined.

***Holotype*** male. “Ecuador. Napo. Km23, | Via Sta. Barbara-La | Bonita. 2400m. | 7–9 April 1986”, “collected by | S.H. McKamey”, and a red “HOLOTYPE | Diestostemma | bicristata | McKamey” (USNM). The left forewing and hind wing are damaged.

#### Measurements (mm).

Total length (from anterior of head to tip of forewings in repose) 13.8; crown length 2.6; transocular distance 2.8; interocular distance 2.0; distance between compound eye and mesal line 1.0; distance between ocellus and mesal line 0.5; pronotal disc maximum width 3.4; pronotal disc maximum length 3.0; forewing length 9.5; length of metathoracic femur 2.4; metathoracic tibia 4.8.

#### Description of the male holotype.

**Figures 1–4.**
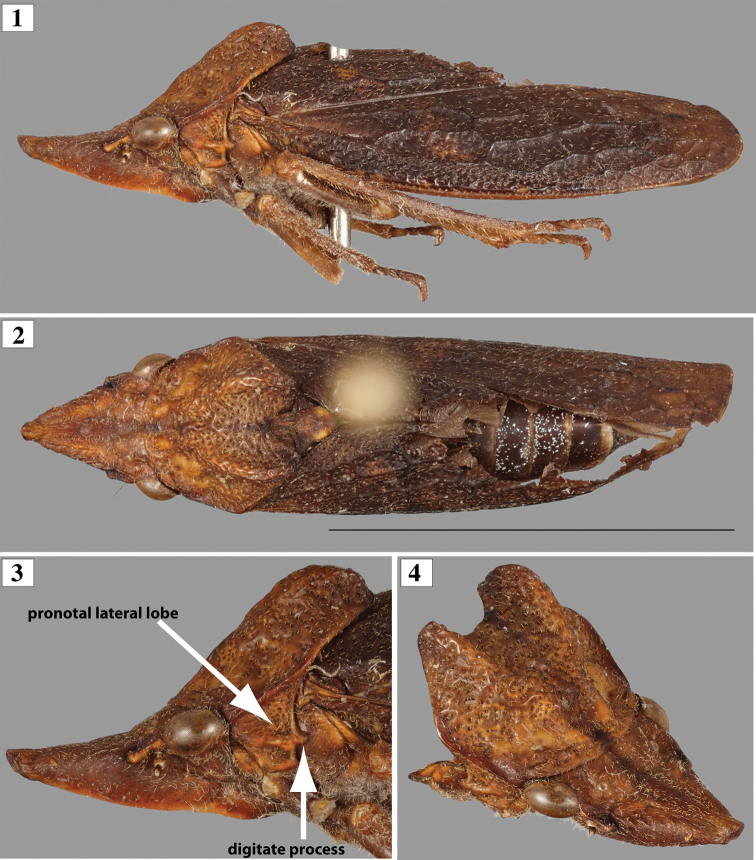
*Diestostemmabicristata*, sp. nov., holotype. **1** Habitus in lateral view (right side, image horizontally flipped) **2** habitus in dorsal view (scale = 2 mm) **3** Head and partial thorax, lateral view **4** head, pronotum, and partial pleura, oblique view. Scale bar: 5 mm.

Body (Figs 1, 2) lacking conspicuous, white powdery brochosomal coat when collected. Head (Figs 3, 4, 6, 7). Crown maximum length 0.9 transocular distance and longer than interocular distance (ratio of 1.3) in dorsal view; anterior margin rounded with small apical, scarlike concavity (Fig. 7); epicranial suture indistinct; frons with deep muscle impressions laterally and planar medially, dorsal surface planar, weakly upturned distally; frontogenal suture extending onto crown to ocellar level. Ocellus located at level of anterior limit of compound eye, distinctly closer to eye than mesal line (ratio of distances between ocellus and eye with eye to mesal line of 0. 39). Epistomal suture indistinct. Clypeus anterior margin in lateral view at level of frons.

Thorax (Figs 1–4, 6). Pronotum maximum width at posterolateral angles 1.1 times wider than transocular distance; maximum length 1.2 times longer than crown length; lateral margins convergent anteriorly; disc sculptured dorsally by punctures and callosities, punctures numerous and closer to each other in posterior half; pair of small transverse anterolateral pits just posterior to anterior margin, followed by smooth polished elevated area with pair of low, parallel median ridges; anteromesal area with elevated polished areas (callosities); posterior two-thirds developed into pair of ridges, gradually higher and more separated posteriorly (Figs 4, 6); posterior margin sinuous with widened W-shaped outline; dorsolateral carina complete; lateral lobe of pronotum punctate, with median ridge, posterior margin projected into short thumb-like process. Mesonotum not punctate; scutellum dorsally smooth, lacking longitudinal carina; high and bluntly rounded until abrupt declivity just before acuminate apex. Forewing (Figs 1, 2) coriaceous; surface strongly punctate, punctures minute distally; venation sclerotized and strongly elevated throughout; basal two-thirds with extra crossveins, distal third weakly reticulate. Hind wing membranous and lacking conspicuous brochosomes. Metathoracic leg with femoral setal formula 2:0:0:0 (AD1 and PD1); tibia with anteroventral row (AV) complete with cucullate (sensu Deitz 1975) macrosetae; anterodorsal (AD) and posteroventral (PV) rows complete with uniform non-cucullate macrosetae; posterodorsal (PD) row with smaller, more closely spaced, uniform, non-cucullate macrosetae; ratio of length of each individual tarsomere by total tarsus length (excluding pretarsus) equal to 0.5, 0.4 and 0.3, respectively.

**Figures 5–12.**
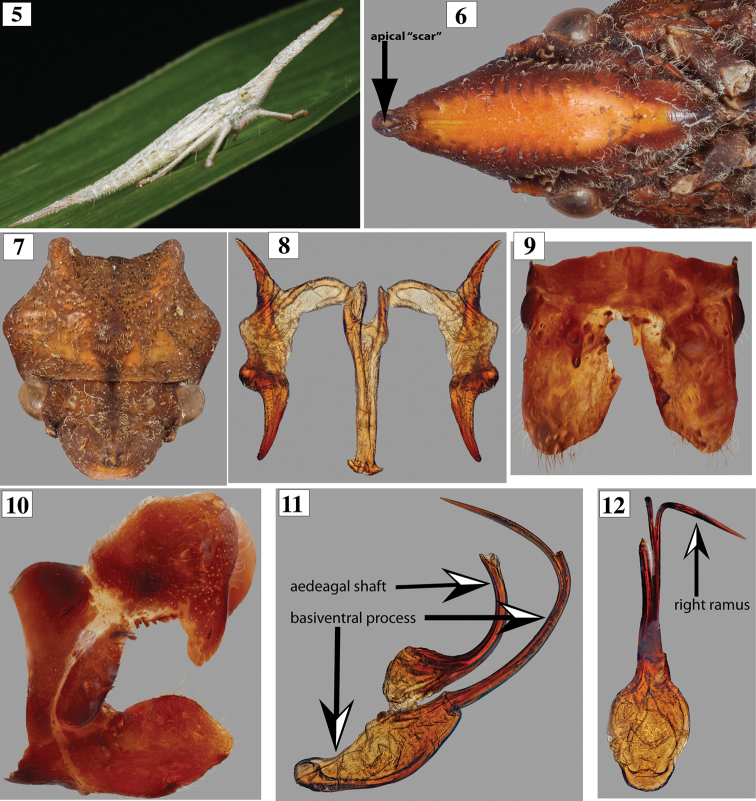
*Diestostemma* spp. **5***Diestostemma* sp., nymph, from Mérida, Venezuela (courtesy of D. Takiya) **6–12***Diestostemmabicristata*, sp. nov., holotype **6** head and partial thorax, ventroanterior view **7** head and pronotum, dorsoanterior view **8** connective and styles, dorsal view **9** pygofer base and subgenital plates, ventral view **10** pygofer and subgenital plate, lateral view **11, 12** aedeagus and basiventral process in lateral and posterior views, respectively (left ramus broken).

Coloration. Head and thorax including legs pale brown except median of frons orange and forewings dark brown and hind wings smoky translucent. Abdomen dark brown.

Male terminalia. Pygofer (Fig. 10) dorsal margin convex, projecting dorsoposteriorly; inner surface without lobe in ventral view; posterior margin slightly concave at middle; microsetae distributed only on posterior lobe surface. Valve, in ventral view, transverse, subrectangular; fused laterally to pygofer lobe; articulated to subgenital plate. Subgenital plate (Fig. 9) 1.4 times longer than wide at base in ventral view; dorsal surface with tooth-like process near outer margin, associated with distal portion of style; posterior margin broadly rounded; microsetae distributed throughout ventral surface, tuft of longer setae at dorsoposterior angle. Style (Fig. 8), in dorsal view, with preapical lobe; apex of apophysis weakly acute, directed posteriorly; ventral margin without preapical dentiform processes. Connective (Fig. 8) 3.9 times longer than maximum width, sublinear posteriorly, narrowing anteriorly with anterior arms weakly separated in dorsal view; stalk much longer than arms. Aedeagus (Figs 11, 12) strongly sclerotized; basally wide, narrowing distally into cylindrical, curved, sickle-shaped shaft in lateral view; shaft posterodistal portion membranous; basiventral process bulbous basally, abruptly constricted adjacent to where aedeagal shaft narrows, extending as narrow single process for half of remaining length before bifurcating and expanding into pair of long needle-like rami; rami of basiventral processes curved anterolaterally, distally divergent in posterior view (left fork broken in holotype).

Female unknown.

#### Distribution.

Known only from the type locality at the Kilometer 23 post along the road between Santa Barbara and La Bonita, at 2400 m elevation, in Napo Province, Ecuador. Biology and ecology unknown.

#### Etymology.

The specific name is feminine and based on the Latin “*crista*,” for crest, in reference to its unique double-crested pronotum.

#### Discussion.

The new species is externally most similar to *D.truncatipenne* Schmidt, which also has an extended vertex and strongly raised forewing veins that are not very reticulate compared to most species of the genus; *D.truncatipenne* has been recorded only from Peru. The aedeagus is most similar to *D.bituberculatum*, which has more abruptly curved basiventral processes; *D.bituberculatum* is recorded from Brazil, French Guiana, and Guyana.

## Supplementary Material

XML Treatment for Diestostemma bicristata
